# Omega-3 fatty acids in first-episode schizophrenia - a randomized controlled study of efficacy and relapse prevention (OFFER): rationale, design, and methods

**DOI:** 10.1186/s12888-015-0473-2

**Published:** 2015-05-02

**Authors:** Tomasz Pawełczyk, Marta Grancow, Magdalena Kotlicka-Antczak, Elżbieta Trafalska, Piotr Gębski, Janusz Szemraj, Natalia Żurner, Agnieszka Pawełczyk

**Affiliations:** Department of Affective and Psychotic Disorders, Medical University of Lodz, ul. Czechoslowacka 8/10, 92-216 Lodz, Poland; Central Teaching Hospital, Medical University of Lodz, ul. Pomorska 251, 92-213 Lodz, Poland; Department of Nutrition Hygiene and Epidemiology, Medical University of Lodz, ul. Jaracza 63, 90-251 Lodz, Poland; Scanlab Medical Diagnostics, ul. Przedzalniana 66, 90-338 Lodz, Poland; Department of Medical Biochemistry, Medical University of Lodz, ul. Czechoslowacka 8/10, 92-216 Lodz, Poland; Clinical Psychology Resident in the Department of Affective and Psychotic Disorders, Medical University of Lodz, Lodz, Poland

**Keywords:** Omega-3 fatty acids, Eicosapentaenoic acid, Docosahexaenoic acid, Schizophrenia first episode, Relapse, Prevention, Randomized controlled trial

## Abstract

**Background:**

Polyunsaturated fatty acid (PUFA) metabolism abnormalities have been long implicated in the etiology of schizophrenia. Although several randomized clinical trials have been carried out to assess the efficacy of omega-3 PUFA as add-on therapy in reducing psychopathology in populations of chronic patients with schizophrenia, only a few concern first-episode schizophrenia. The majority of these studies used a 12-week intervention based on ethyl-eicosapentaenoic acid (ethyl-EPA), however, with conflicting results. An intervention based on docosahexaenoic acid plus EPA has not been used in first-episode schizophrenia studies so far. No add-on supplementation studies have been carried out in medicated first-episode schizophrenia patients to assess the efficacy of omega-3 PUFA in preventing relapses.

**Methods:**

A randomized placebo-controlled one-center trial will be used to compare the efficacy of 26-week intervention, composed of either 1320 mg/day of EPA and 880 mg/day of DHA, or olive oil placebo with regard to symptom severity and relapse rate in first-episode schizophrenia patients. Eighty-two patients (aged 16–35) will be recruited for the study. Eligible patients will be randomly allocated to one of two intervention arms: an active arm or a placebo arm (olive oil). The primary outcome measure of the clinical evaluation is schizophrenia symptom severity measured by the Positive and Negative Syndrome Scale (PANSS). Other outcomes include depressive symptoms, patient functioning and the level of insight. Correlates of change measured during the study will include structural brain changes, oxidative stress and defense, as well as neuroplasticity indicators. Metabolic syndrome components will also be assessed throughout the study.

**Discussion:**

By comparing 26-week administration of EPA + DHA or (placebo) olive oil as add-on therapy in reducing symptom severity and one-year relapse rate in patients with first episode schizophrenia, it is intended to provide new insights into the efficacy of omega-3 PUFA and correlates of change, and contribute to the improvement of mental health care for individuals suffering from schizophrenia.

**Trial registration:**

This study has been registered at Clinical Trials.gov with the following number: NCT02210962.

## Background

### Polyunsaturated fatty acids in schizophrenia

Schizophrenia is a neurodevelopmental and neurodegenerative disorder displaying disturbance in multiple neurotransmission pathways. Since polyunsaturated fatty acids (PUFA) are essential for normal neurodevelopment, disturbances of PUFA metabolism may be involved in the etiology of neurodevelopmental disorders like schizophrenia [1]. PUFA are the major constituents of cell membrane phospholipids and take part in numerous biological processes including receptor binding, neurotransmission, signal transduction and the synthesis of active metabolites (i.e. eicosanoids), which control inflammation, oxidative stress and defense processes, among others. PUFA are abundant in the central nervous system, where they are the main constituents of neuronal membrane phospholipids. Long-chain PUFA, i.e. docosahexaenoic acid (DHA) and arachidonic acid (AA) are incorporated in Sn-2 position of phospholipids. DHA and AA are released from membrane phospholipids by phospholipase A2, and their derivatives take part in neuronal impulse transmission. Neuronal membranes are rich in DHA, which is essential for normal brain development [2]. The DHA/AA (n-3/n-6) ratio is important in the maintenance of an appropriate level of biological membrane fluidity, which is in turn, essential for ion channel function, membrane receptor activity and the release of neurohormones, i.e. the signaling processes of every cell. Numerous neurotransmission pathways have been found to be dysfunctional in schizophrenia, which raises the probability that membrane phospholipids that modulate the activity of both receptors and are involved in signal transduction may be part of the etiology of schizophrenia.

PUFA metabolism disturbances have repeatedly been described in patients with schizophrenia. Deficiencies of PUFA were described in red blood cell (RBC) membranes of patients with schizophrenia [3-6]. PUFA RBC concentration is known to reflect its concentration in the brain in adult humans [7]. Disturbances in PUFA metabolism have been shown to exist even at early stages of schizophrenia prior to initiation of antipsychotic therapy [3] and in individuals at ultra-high risk of psychosis [8]. A recent meta-analysis confirms the presence of n-3 and omega-6 (n-6) PUFA abnormalities in antipsychotic-naive, antipsychotic-free schizophrenia patients and in patients currently treated with antipsychotics [9]. Post-mortem brain studies have revealed decreased levels of PUFA, especially docosahexaenoic acid and arachidonic acid, in the frontal lobes of schizophrenia patients. PUFA deficiencies in neuronal membranes can be the result of the increased release of PUFA from membrane phospholipids, or its decreased incorporation within them. Both processes were found to be defective in schizophrenia patients.

Since PUFA cannot be effectively synthesized in humans, decreased incorporation may be the result of dietary deficiencies. One of our recent studies identified differences in declared PUFA consumption between UHR individuals who converted to full-threshold psychosis and who did not develop psychotic disorder (article submitted for publication). Differences were also found in declared PUFA consumption between healthy controls (HC), UHR individuals and patients with first-episode schizophrenia (FES). The PUFA consumption patterns of UHR individuals were comparable with those of FES patients and were significantly different from HC subjects: UHR and FES groups declared significantly higher consumption of omega-6 fatty acids than the HC group.

### Membrane phospholipids hypothesis of schizophrenia

The repeated observation of lipid metabolism dysfunctions in patients with schizophrenia led to the formulation of the Membrane Phospholipid Hypothesis of Schizophrenia by David Horrobin [10], according to which, cellular PUFA abnormalities can lead to schizophrenia in susceptible individuals. A number of pathophysiological changes observed in schizophrenia can be related to PUFA metabolism: eg. decreased membrane fluidity [11], altered monoaminergic neurotransmission [12-14], production of proinflammatory cytokines, increased oxidative stress damage to the neuronal membrane and reduced anti-oxidative defense [15]. PUFA depletion in schizophrenia can be a result of increased phospholipase A2 activity and oxidative damage, which together lead to increased release of PUFA from neuronal membranes [16]. Phospholipase A2 (PLA2) gene polymorphisms have been associated with susceptibility to schizophrenia [17,18] and antipsychotic medications have been found to decrease PLA2 expression in patients with schizophrenia [19]. Another mechanism influencing PUFA levels in the cell membranes of schizophrenia patients is decreased incorporation of PUFA into membrane phospholipids due to lipoprotein lipase dysfunction and inadequate synthesis of long-chain PUFA from their essential precursors: linoleic acid (LA) for n-6 PUFA and alpha-linolenic acid (ALA) for n-3 PUFA [20].

### Efficacy of n-3 PUFA in schizophrenia

#### Supplementation studies in schizophrenia

Observations of PUFA metabolism dysfunctions in schizophrenia patients at different stages of disease together as well as those with PUFA dietary deficiencies prompted intervention trials. Seven randomized controlled trials (RCTs) comparing n − 3 PUFA eicosapentaenoic acid (EPA) with placebo as supplemental treatment to antipsychotics in schizophrenia have been reported. Two of these studies report that EPA had a positive effect on primary efficacy [21,22], and all but one [23] of the others reported at least some beneficial effect on secondary outcomes [21,24-26]. One study on 80 first-episode patients over 12 weeks found EPA to decrease time to response in patients with non-affective psychosis, and showed a 20% reduction in antipsychotic use in the EPA augmented group [26]. A recent meta-analysis of RCTs revealed no beneficial effect of EPA augmentation on symptom severity in schizophrenia. However, no conclusion could be drawn regarding the medium- to long-term effects of EPA in schizophrenia, particularly regarding relapse prevention in the early course of psychotic disorders [27]. Moreover, a 12-week RCT conducted in individuals at high clinical risk of schizophrenia provides preliminary evidence that intervention composed of 1.2 g of PUFA (i.e. EPA + DHA) could prevent transition to first-episode psychosis.

Supplementation with n-3 PUFA may demonstrate higher efficacy in populations of individuals at high-risk of psychosis and early psychosis than in patients with chronic schizophrenia. A recent study notes that PUFA metabolism disturbances are present relatively early in the course of schizophrenia and diminish over time as the disease progresses, and that treatment with antipsychotic medications (aripiprazole and risperidone) was able to correct aberrant PUFA levels in first-episode schizophrenia patients, but not in a group of patients with chronic schizophrenia [28]. This suggests that intervention with n-3 PUFA can be more effective when administered early in the course of disease.

### PUFA and the reduced risk of such adverse effects as tardive dyskinesia and metabolic side effects associated with antipsychotics

A growing body of evidence suggests that n-3 PUFA may play a role in reducing the intensity of adverse effects associated with antipsychotics. Antipsychotic-induced dyslipidemia was successfully treated with n-3 PUFA supplementation [29]. Short term (6 weeks) n-3 supplementation was able to modulate cardiometabolic risk factors in patients treated with olanzapine and either valproate or lithium [30]. Berger et al. report that ethyl-EPA supplementation used as add-on therapy in patients with schizophrenia was associated with a 20% reduction in the use of antipsychotics, reduced percentage of patients experiencing the extrapyramidal side effects, constipation and the sexual side effects of antipsychotic therapy [26]. Additionally, n-3 PUFA supplementation has been shown to play a possible role in reducing the risk of tardive dyskinesia [22], however, these observations are not consistent [25]. Reduction of motor side effects of antipsychotics have also been observed in animal models [31-33]. Reduction in the intensity of side effects associated with n-3 supplementation can increase adherence to antipsychotics and in turn, influence the severity of symptoms and the relapse rate in first episode schizophrenia.

### Relapse in schizophrenia

#### Relapse in schizophrenia

It is estimated that up to 90% of individuals developing first-episode schizophrenia achieve remission from symptoms in the first 12 months of therapy [34] but recovery defined as attainment of both functional and symptomatic remission for 2 years [35] is achieved in only 10-20% of patients [36]. This is partly because a great number of patients will experience a relapse of psychotic episodes during the first years following diagnosis. Longitudinal studies find that about one third of patients will have experienced a relapse before 1 year after recovery, and two thirds before 2 years [37,38]. Seventy to eighty percent of patients experience relapse within three to five years [39]. There is some evidence that with recurring episodes of psychosis, the time to obtain response increases and the proportion of patients achieving remission diminishes [40]. Psychotic relapse and incomplete recovery can lead to the chronic phase of the disease. Neurodegeneration and deterioration occurs mainly in the first 5–10 years of illness. The long-term outcome of the disease may be associated with the number of relapses and the quality of remission achieved [40]. These observations have generated increased interest in relapse prevention in schizophrenia and have prompted repeated attempts to understand the risk factors responsible for recurrence of the psychotic exacerbation.

#### Relapse risk factors

A systematic review based on a meta-analysis of clinical trials revealed that the risk of relapse in schizophrenia decreases with antipsychotic medication use [41], and the main risk factor for relapse is antipsychotic therapy discontinuation [38,40]. The main reason for antipsychotic non-adherence are safety and tolerability issues, as well as lack of insight associated with psychosis. Although second generation antipsychotics are frequently used in treatment of patients diagnosed with schizophrenia, they are associated with a high risk of adverse cardiometabolic effects such as weight gain, glucose intolerance and diabetes, and lipid profile abnormalities. A recent study has found that both duration of relapse and the intensity of treatment with antipsychotics may be associated with brain volume loss [42]. Andreasen et al. found that the duration of relapse was closely related to loss of brain tissue over time in multiple brain regions, including total cerebral volume loss, as well as loss in subregions, particularly the frontal lobes. They report that relapse duration and treatment intensity both contribute to brain tissue loss, but while treatment effects are more diffusely distributed, the relapse effects are most strongly associated with changes to the frontal lobe tissue. The authors conclude that relapse prevention should be sustained using the lowest possible medication dosages to control the symptoms.

#### n-3 PUFA in relapse prevention

To date, only one study of the use of n-3 PUFA in relapse prevention has been reported. Emsley et al. recently published the results of a study assessing the efficacy of intervention composed of 1 g of DHA and 2 g of EPA plus 300 mg of the antioxidant alpha-liopic acid (LA) or placebo in patients diagnosed with schizophrenia, schizoaffective disorder or schizophreniform disorder meeting the criteria of relapse in whom antipsychotic therapy was tapered and discontinued according to the current guidelines. The study was terminated prematurely due to the severity of some of the relapse episodes, as well as the high relapse rates in both treatment groups: 90% and 75% in active and placebo groups respectively. The authors did not observe significant differences in relapse rates between the study arms. The study concludes that n-3 PUFAs + alpha-LA does not appear to be a suitable alternative to maintenance antipsychotic treatment in relapse prevention. The authors argue that on the basis of their findings, the current treatment guidelines suggesting antipsychotic discontinuation 2–3 years after the first psychotic episode should be revised [43].

### Rationale for the study

Hence, there is a growing body of evidence based on animal models, *in vitro* and observational studies, as well as from randomized clinical trials, which suggests that n-3 PUFA plays a role in the pathophysiology of schizophrenia, and that supplementation with these essential compounds may be worth consideration, especially at early stages of the disorder. N-3 PUFA supplementation was found to be effective in reducing the risk of transition into psychosis in individuals at ultra-high risk of psychotic disorders. Data from basic studies indicates that PUFA metabolism impairments are present at early stages of schizophrenia. However, meta-analyses of RCTs do not indicate that n-3 PUFA may be efficacious in reducing the severity of symptoms in chronic schizophrenia patients: antipsychotic medications are still the mainstay of schizophrenia therapy.

The number of schizophrenia patients treated with antipsychotic polypharmacotherapy is increasing [44], despite there being insufficient evidence from well-designed clinical trials and in spite of accumulating data implicating several serious adverse effects and increased health costs associated with antipsychotic polytherapy [45]. Despite increasing antipsychotic usage, more than 80% of patients experience relapse within the first 5 years following the first psychotic episode and the majority of deterioration takes place during the first few years after diagnosis. Unplanned antipsychotic discontinuation was shown to be the main risk factor for relapse in schizophrenia [40]. However, Andreasen et al. report brain volume loss to be associated not only with the duration of psychosis, including relapses, but also with the intensity of antipsychotic utilization [42]. Hence, it is particularly important that the smallest possible doses of antipsychotics are used to reduce the duration of psychotic episodes in schizophrenia, including relapses. To this end, n-3 supplementation was shown to be safe and well tolerated [46], and can allow reduced doses of antipsychotic medications to be used to achieve and sustain remission in schizophrenia [26].

Most previous studies assess the short-term efficacy of n-3 fatty acids in the exacerbation of chronic schizophrenia, but few of them have been conducted in a population of first-episode schizophrenia patients. The intervention period was 8 weeks in the majority of studies. It is well established that the period needed to refill the n-3 fatty acid store in the human body, and hence to achieve maximum metabolic effects, is about 3–4 months. Most studies assess an intervention composed of only EPA, one study compared EPA and DHA in two separate study arms and only one study used a mixture of EPA + DHA. The only study that reports an intervention composed of EPA + DHA was conducted in ultra-high risk patients.

It is important to underline that most reported studies were carried out in chronic schizophrenia patients, while only two of them supplemented first-episode patients. Moreover, the majority of the RCTs conducted so far are based on ethyl-eicosapentaenoic acid (ethyl-EPA) and most interventions were short term, i.e. 8–12 weeks. DHA is an important constituent of neuronal membranes, which have an important role in brain development and function. The DHA molecule itself has unique structural properties that appear to provide optimal conditions for a wide range of cell membrane functions. Its presence in the neuronal membranes provides a biochemical and biophysical basis for membrane fluidity and neurotransmission. In addition, it was recently found to be a source of two important endogenous neuroprotective compounds: protectins and resovlins.

One rudimentary source of DHA is marine algae, but it can be found in a concentrated form in fish and marine oils. Mammalian cells lack the specific enzymes required for the *de novo* synthesis of alpha-linolenic acid (ALA), the precursor for all n-3 fatty acid syntheses. The endogenous synthesis of DHA from ALA in humans is much more limited than previously assumed, and low consumption of fish and marine oils can lead to DHA deficiencies. In addition, the excessive consumption of omega-6 fatty acids in the modern Western diet further displaces DHA from membrane phospholipids. An emerging body of research suggests that DHA plays a unique role in neurodevelopment [47,48] and the prevention of neuropsychiatric [49] and neurodegenerative disorders [50,51].

Hence, it is planned to conduct a long-term randomized, placebo-controlled, single site study of a EPA + DHA intervention in first-episode schizophrenia patients. The efficacy of EPA + DHA added-on to antipsychotics in reducing psychopathology and the relapse rate in early schizophrenia have not yet been studied systematically. The OFFER study aims to systematically evaluate the long-term efficacy of n-3 PUFA in reducing psychopathology and preventing relapse in patients with first-episode schizophrenia.

### Main research questions and hypotheses

The following research questions were formulated:

1. Is supplementation with n-3 fatty acids added to antipsychotic medications an efficacious intervention for patients in the acute phase of first-episode schizophrenia?
2. Can add-on therapy with n-3 fatty acids initiated in the acute phase of disease be efficacious in reducing the one-year relapse rate in first-episode schizophrenia.
3. Is add-on therapy with n-3 PUFA related to changes in cognitive performance of first-episode schizophrenia patients?
4. What are the biochemical and structural brain correlates of n-3 PUFA add-on therapy in first-episode schizophrenia patients? Specifically, are oxidative stress and defense as well as brain plasticity mechanisms involved? Is add-on n-3 PUFA supplementation associated with structural brain changes concerning gray and white matter, such as cortical thickness and fiber density, axonal diameter or myelination?

## Methods

### Study design

The study will be based on a randomized, double-blind, placebo-controlled, parallel-group single-center augmentation trial (RCT) of either 2.2 g per day of n-3 PUFA, or olive oil placebo, added on to an adjustable dose of antipsychotic medication. The background antipsychotic therapy will be chosen and titrated according to the Polish standards of pharmacotherapy of mental disorders [52]. The use of benzodiazepines (diazepam, lorazepam, alprazolam, clonazepam), chlorprothixene, zuclopenthixol acetate will be allowed if control of behavioral symptoms is indicated. Zolpidem and zopiclone will be used in patients complaining of insomnia. For patients presenting significant depressive symptoms the use of selective serotonin reuptake inhibitors will be allowed. For all participants, the protocol allows for treatment of emergent extrapiramidal side effects with biperiden and treatment of akathisia with propranolol, lorazepam, or clonazepam. Using PUFA supplements other than contained in study medication will not be permitted throughout the study.

### Participant sample

The intended study population is composed of inpatients admitted to the Psychiatric Clinics of the Central Teaching Hospital, Medical University of Lodz. Patients will be enrolled consecutively as they are admitted to the hospital. Eligible patients will be (1) aged 16–35, (2) diagnosed with first-episode schizophrenia according to the International Classification of Diseases 10th version (ICD-10), which is an obligatory classification of mental disorders in Poland, (3) currently psychotic as reflected by the presence of at least one psychotic symptom daily for more than one week (delusions, hallucinations, disorders of thought form other than simple acceleration or retardation, and disorganized, bizarre, or markedly inappropriate behavior). Diagnosis will be confirmed by the mini neuropsychiatric interview plus (MINI plus) [53]. Patients will be excluded if more than two years have passed since the onset of positive symptoms, if the patient have bleeding disorders, or have been taking fish oil supplements or anticoagulants for any reason, were diagnosed with drug-induced psychosis, first-episode mania or organic disorders presenting with psychotic symptoms, if the patient has a history of intellectual disability, or a history of head injury with loss of consciousness, or any acute or unstable medical condition.

The trial procedures will be explained verbally and in writing to all eligible patients. All participants will provide written informed consent prior to study enrollment. The study will be conducted in accordance with the Declaration of Helsinki and the study protocol has been approved by the Ethics Committee of the Medical University of Lodz.

### Power calculation

Previous studies have suggested medium effect sizes in favor of n-3 fatty acids [21,22]. On the basis of previous studies, we assume that there will be a nontrivial correlation between baseline and outcome scores at follow-up, and it is hypothesized that baseline scores will explain 25% of the variation in outcome measures. Power calculation revealed that 36 participants per study arm will result in 80% power to detect medium effect size (0.3) at a significance level of 0.05 (two sided). Assuming an attrition rate of 15%, we aim to enroll 82 patients.

### Randomization and procedure

The study medication (omega-3 PUFA and placebo) will be provided by Marinex International Sp. z o.o. and shipped from Scandinavian Laboratories, Inc. Mt. Bethel, PA, USA. It will be sent to the store of the Central Teaching Hospital of the Medical University of Lodz, Poland, including a randomization code list and sealed envelopes for unblinding in case of a serious adverse event. Patients will be allocated the lowest available number of the randomization code list, and the Clinical Trial Store will dispense bottles of study medication according to the allocated number. Each bottle contains capsules of study medication, i.e. either 1320 mg/day of EPA plus 880 mg/day of DHA or an equal amount of an olive oil placebo. Both placebo and active capsules contain an antioxidant, i.e. 0.2% alpha-tocopherol (vitamin E). The placebo contains also a scant amount of fish oil to provide a comparable taste of the different capsules. The key to the randomization list will remain with Marinex International Sp. z o.o. until the last study participant completes the trial. Both the patients and the study staff responsible for study assessments will remain blind to the status of the patients throughout the study. Participants will be assessed by a registered dietician (ET) and advised to adhere to a balanced diet for the duration of the study.

### Interventions

#### Rationale for intervention arms

Early studies of red blood cell membranes in patients diagnosed with schizophrenia found deficiencies of both n-3 and omega-6 fatty acids, especially arachidonic acid (AA) and docosahexaenoic acid (DHA). Similar findings were found by post mortem studies of the frontal lobes of patients. The formulation of the Membrane Phospholipid Hypothesis of schizophrenia resulted in a number of double blind, placebo controlled intervention studies in patients with schizophrenia. Initial studies were quite small and were based on omega-6 PUFA, mainly gamma linoleic acid, and the results were generally negative [54]. Later, other n-3 PUFA were used in a series of intervention trials.

A double blind placebo-conntrolled study by Peet et al. [21] assessed the efficacy of add-on 12 week supplementation with 2 g/day EPA enriched oil, 2 g/day DHA enriched oil or a corn oil placebo in reducing the intensity of symptoms in patients with chronic schizophrenia. At the end of the trial, the PANSS score was significantly lower in the EPA-supplemented patients than in the placebo group. No significant differences were found between the DHA enriched oil group and the placebo group. The same group of researchers also tested the efficacy of 12-week supplementation with 2 g/d EPA enriched oil as a sole treatment for new or relapsed cases of schizophrenia who were not treated with antipsychotics. After a 3-month intervention period, the EPA-treated group had significantly lower PANSS scores than the placebo group. Moreover, significantly fewer EPA group patients required treatment with antipsychotics at the end of the study and the length of antipsychotic therapy was significantly shorter in the EPA supplemented group than the placebo corn-oil group.

Peet and Horrobin [24] followed up these early studies with a dose-ranging placebo-controlled study of 1, 2 or 4 g/day of ethyl-EPA or liquid paraffin-placebo as add-on therapy of chronic schizophrenia patients. The results indicate that a dose of 2 g/day of ethyl-EPA used for 12 weeks significantly reduced psychopathology in comparison with placebo but only in patients treated with atypical antipsychotic - clozapine. Doses of 1 and 4 g/day for 12 weeks were not found efficacious.

Three other teams of researchers have assessed EPA supplementation as add-on therapy in patients with chronic schizophrenia using double-blind placebo controlled trials. Fenton et al. [23] observed no significant differences in the PANSS scores of chronic schizophrenia patients treated with either mineral oil placebo or 3 g/day ethyl-EPA for 16 weeks. However, RBC membrane PUFA level significantly increased in both groups, which could suggest that the participants consumed n-3 PUFA rich food or supplements during the trial. Another trial by Emsley et al. [22] noted significantly higher reduction of PANSS scores in chronic medicated schizophrenia patients receiving 12-week interventions of 3 g/day of ethyl-EPA then those given paraffin oil placebo. The highest reduction was observed in patients treated with typical antipsychotics.

Negative findings have also been found in chronic schizophrenia patients. Norwegian researchers [55] report generally negative results for 2 g/day of ethyl-EPA, vitamin C (1000 mg/day) and vitamin E (364 mg/day) for 16 weeks as add-on therapy of psychotic exacerbation in chronic schizophrenia patients. The authors conclude that given separately during an acute episode, EPA and vitamins E + C increased the severity of psychotic symptoms in patients with low red blood cells (RBC) levels of PUFA. When combined, the effect of these agents on symptom severity was not significantly different from placebo. No significant effects were observed for combined EPA and vitamin C + E administration on PANSS score in high-RBC PUFA level individuals.

Negative findings were also reported for the effect of n-3 PUFA in first-episode schizophrenia patients. Another group of researchers [26] assessed the effect of 2 g/day of ethyl-EPA add-on therapy carried out for 12 weeks on symptom severity in early psychosis. The study results did not demonstrate a sustained symptomatic benefit of ethyl-EPA in early psychosis, but ethyl-EPA was able to accelerate treatment response and improve the tolerability of antipsychotic medications. The authors argue that a ceiling effect may have been responsible for the negative results of the study, since a high proportion of first-episode patients already achieve symptomatic remission with antipsychotic medication alone.

Finally, n-3 PUFAs were assessed in individuals at ultra-high risk (UHR) of psychosis. A randomized placebo-controlled clinical trial by Amminger at al [56] indicates that 12-week supplementation with 1.2 g/day n-3 PUFA (i.e. 700 mg of EPA and 440 mg/day of DHA) is effective in preventing transition to full-threshold psychosis in UHR individuals.

To sum up, the results of clinical trials of n-3 fatty acid supplementation in chronic schizophrenia are inconsistent. EPA was mainly used in the trials. The majority of positive results were obtained with 12-week EPA supplementation at daily doses of 2 and 3 g [21,22,24]. DHA alone was used in one study conducted by Peet [21] and the results were negative. One study based on first-episode schizophrenia did not identify any positive effects of EPA on psychopathology, possibly due to the ceiling effect [26]. A 12-week supplementation with 1.2 g/daily of EPA + DHA was found efficacious in preventing transition to full-threshold psychosis in individuals with clinical high risk of schizophrenia development [56].

The rationale for using EPA supplementation to study efficacy in early schizophrenia and relapse prevention is mainly based on the findings of the chronic schizophrenia studies described above. The observation that EPA + DHA add-on therapy could prevent psychotic disorder development in UHR individuals supports an intervention comprising both EPA and DHA. The rationale for using DHA supplementation is based mainly on evidence accumulating from basic, animal and human studies, indicating that the DHA molecule has unique structural properties which appear to provide optimal conditions for a wide range of cell membrane functions. This has particular implications for grey matter, which is membrane-rich tissue and abundant in DHA. A recently identified important metabolic role played by DHA is the precursor for resolvins and protectins, both of which have anti-inflammatory properties [57] and provide neuroprotection against oxidative stress and apoptosis [58]. Oxidative stress and neuroinflammation has long been implicated in the pathogenesis of schizophrenia. Since poor cognitive function was found to be a risk factor for relapses of schizophrenia, it is significant that it can be influenced by DHA. DHA was found to improve cognition in healthy individuals [59,60] and in animal models [12,61], it has been found to be involved in neurogenesis and neuroplasticity [62] and is thought to be essential for neurodevelopment [48].

EPA and DHA supplementation was found to be efficacious in an open label study of schizophrenia patients treated with haloperidol [63]. Only one study addresses the efficacy of n-3 PUFA administration in reducing relapse rate in schizophrenia [43]. An intervention based on 2 g of EPA plus 1 g of DHA and 300 mg of antioxidant alpha-lipoic acid daily was used as the sole treatment in stable schizophrenia patients whose antipsychotic therapy was discontinued according to the current guidelines. The results of the study were negative. No significant differences in relapse rate were found between the active and placebo groups.

It is intended to use an intervention based on EPA and DHA at a ratio of 1.5:1 by weight in the proposed OFFER study. This form of intervention has not been used so far in first-episode schizophrenia patients. In addition, trials using only EPA supplementation have returned inconsistent findings, and the RCT carried out by Amminger et al. discussed above has already demonstrated the efficacy of EPA + DHA in reducing the risk of conversion to psychosis in individuals at ultra-high risk. Hence, an OFFER study based on a long-term intervention comprising both EPA and DHA as add-on therapy appears to offer a great deal of potential.

### Doses

The study participants will be supplemented with 1320 mg/day of EPA and 880 mg/day of DHA. Higher doses of EPA and DHA will be used in the OFFER trial than in the study led by Amminger et al., who assessed transition to psychosis in individuals with high clinical risk. It is assumed that the higher level of psychopathology present in first episode schizophrenia patients reflects higher PUFA metabolic abnormalities, which require higher doses of n-3 PUFA for normalization. The doses of EPA and DHA intended to be used in the present study have been found to be well tolerated and safe in earlier studies of patients diagnosed with schizophrenia [21].

### Control condition

Olive oil was chosen as placebo because it contains mainly monounsaturated fatty acids and only small amounts of unsaturated and polyunsaturated fatty acids. Previous studies used placebo composed of liquid paraffin [22,24] and coconut oils [56], which are composed mainly of unsaturated fatty acids. Since one of the secondary outcome measures in the OFFER study was a change in the metabolic profile, an oil with low unsaturated fatty acid content was chosen in order to avoid bias. As the corn oil used in previous studies [21] contains large quantities of omega-6 PUFA which negatively influences n-3 PUFA metabolism, it could influence the main outcome by increasing the effect of the intervention. However, since olive oil contains relatively small amounts of linoleic acid (20:4, omega-6), and the fact that it is a common constituent of the Polish diet, it is a more suitable placebo. In addition, its familiarity as an ingredient has two further benefits: firstly, it could reduce the risk of not giving consent, thus facilitating recruitment by reducing patient concerns, and secondly, it may reduce the non-compliance and attrition rates in the study. Moreover, it could be unethical to use placebo composed of mainly monounsaturated fatty acids in FES patients, who are at risk of developing hyperlipidemia, especially when treated with second generation antipsychotics.

### Duration of intervention

The intervention period in the majority of the previous studies assessing the efficacy of n-3 PUFA in schizophrenia lasted 8 to 12 weeks. No longer term trials were conducted. The present study is planned to last 52 weeks and will be composed of two phases: intervention and follow-up. The intervention period is designed to be continued for 26 weeks. Participants will be observed for the 26 weeks following the intervention phase.

### Medication adherence

For all patients, parent/patient self-report adherence data will be collected regarding intervention and all psychotropic medications at each medication visit. For participants receiving study medication (i.e., n-3 PUFA or placebo), information will also be collected by pill count at all medication appointments.

### Clinical outcome measures

#### Measures and assessment of outcomes

Clinical scales will be used to assess several domains of symptoms and patient functioning. The severity of schizophrenia symptoms will be measured using the Positive and Negative Syndrome Scale [64], which is composed of 30 items scored 1–7, with a total score ranging from 30, indicating an absence of psychopathology, to 208. PANSS is a reliable and valid scale commonly used to score symptom severity in schizophrenia [65].

Depressive features will be measured by the Calgary Depression Scale for Schizophrenia (CDSS) that was developed specifically for schizophrenia patients [66]. It is an observer-rated scale consisting of 9 items, each with 4 anchor points. The validity and reliability of the scale was assessed in adults [67] as well as in adolescents [68].

Patient functioning status will be measured by means of Global Assessment of Functioning (GAF) scale. The GAF is a method for representing a clinician’s judgment of a patient’s overall level of psychosocial functioning [69]. The GAF requires a clinician to make an overall judgment about a patient’s current psychological, social, and occupational functioning. In the DSM-IV, this rating is given on a scale from 1 to 100, with ratings of 1 to 10 indicating severe impairment and ratings of 91 to 100 indicating superior functioning.

The Clinical Global Impressions scale will be used to measure symptom severity (CGI-S) and the range of improvement (CGI-I). CGI-S and CGI-I scales are commonly used to assess efficacy of treatment in patients with mental disorders [70]. The level of insight will be assessed with the “My thoughts and feelings” questionnaire, which was validated in population of Polish patients with schizophrenia [71].

All the raters have undertaken intensive training in the scoring methods used in the study as part of the first Polish program for people at high clinical risk of psychosis development (PORT), which is described in detail elsewhere [72]. The raters achieved good reliability scores (coefficient of agreement >0.82) and were all within 20% of the standard scores.

### Primary outcome measure

The primary outcome measure will be the efficacy of n-3 PUFA in reducing psychopathology in first-episode schizophrenia. The Positive and Negative Syndrome Scale [64] will be used to assess the efficacy of EPA + DHA supplementation in reducing symptom severity in first-episode schizophrenia after 8 and 26 weeks of supplementation. The main outcome measure will be the change in symptom severity from baseline to week 26. Baseline PANSS total score will be subtracted from PANSS score obtained after 26 weeks, resulting in the degree of change observed in the study.

Another way of assessing the efficacy of n-3 PUFA in reducing symptom severity in first-episode schizophrenia patients will be the comparison of the number of responders in each intervention group: 25% and 50% decreases in PANSS total score are considered cut-offs for clinically significant symptom reduction and indicates minimal and great improvement [73]. The percentage change of scores will be calculated according to Leucht et al. [73], based on the principle that a patient with no psychopathological symptoms scores 1 in all PANSS scale items. The formula for calculating change will be as follows: [(Pt2 – N) – (Pt1 – N)/(Pt1 – N)]•100, where Pt1 is the PANSS subscale score at the beginning of the study, Pt2 is the value at the end of the study and N is the number of PANSS subscale items. N = 7 for PANSS positive and negative subscales, N = 16 for the general psychopathology PANSS subscale and N = 30 for the total PANSS score. Patients who achieve at least a 25% reduction in the total PANSS score will be regarded as responders. Those who achieve a 50% decrease will be regarded as much improved.

### The first secondary aim

The secondary aim of the study is to assess whether 26 weeks intervention with 2.2 g of marine long-chain n-3 polyunsaturated fatty acids initiated in the acute phase of first episode schizophrenia can prevent relapse during one year observation. Schizophrenia relapse will be operationally defined as meeting any of one or more of the following specified individual criteria given by Csernansky et al. [74]: (a) a 25% increase in PANSS total score; (b) deliberate self-injury; (c) emergence of clinically significant suicidal or homicidal ideation; or (d) violent behavior resulting in significant injury to another person or significant property damage; (e) admission to psychiatric inpatient clinic due to exacerbation. Since no common agreement exists regarding the relapse criteria, the most commonly used criteria were chosen on the basis of a recent meta-analysis [75]. The presence of relapse will be assessed during all study visits except for the first. The chosen study design concerning relapses is more pragmatic than explanatory and is used to reflect the frequent clinical observation that patients treated for first-episode schizophrenia achieve partial symptomatic remission with persistence of certain symptoms after the acute phase of the illness.

### The second secondary aim

Other secondary clinical outcome measures include the intensity of depressive symptoms, patient functioning, symptom severity (CGI-S), the range of improvement (CGI-I), and the level of insight.

Patients will also undergo dietary assessments at the beginning of the study. This will enable control of that possible confounder at the level of statistical analysis.

To address the third research question about the correlates of the observed change, a number of assessments are planned. Structural changes of the brain, i.e. cortical thickness and fractional anisotropy of the white matter bundles, will be measured using magnetic resonance imaging with tensor diffusion. The level of PUFA metabolites (mainly prostaglandins E2 and D2) will be measured indirectly by using a niacin flush skin test (NFST) described in detail elsewhere by Smesny et al. [76] Oxidative stress and defense will be measured by the total oxidative plasma capacity and 8-epi-alpha-izoprostanes. Neuroplasic changes of the brain will be assessed indirectly by measuring the plasma level of brain-derived neurotrophic factor (BDNF).

Metabolic syndrome components, i.e. weight, body mass index (BMI), waist circumference, serum cholesterol concentration (total, LDL, HDL), serum triglyceride level, systolic and diastolic blood pressure will also be measured during study visits. Descriptive variables will include socioeconomic and demographic variables, variables concerning psychiatric therapy and symptom duration, as well as medical history.

### Study visits

A total of 9 visits will be conducted, including a baseline visit preceding commencement of study medication, followed by 8 follow-up assessments at weeks 1, 2, 4, 6, 8, 16, 26, 52. Clinical assessments and measurements comprising PANSS, CDSS, CGI-S, CGI-S, GAF, Questionnaire of Insight, and metabolic syndrome components, i.e. weight, body mass index (BMI), waist circumference, serum cholesterol concentration (total, LDL, HDL), serum triglyceride level, systolic and diastolic blood pressure will be carried out during the study visits. Correlates of change will also be assessed, i.e. structural brain changes assessed by means of MRI and biochemical measures of oxidative stress and defense, as well as plasma BDNF concentration. The complete schedule of assessments planned in the OFFER trial is presented in Table 1.

**Table 1:** **Schedule of the study visits and planned assessments in OFFER trial**

**ID**	**Variable**	**Study visits**	**V1**	**V2**	**V3**	**V4**	**V5**	**V6**	**V7**	**V8**	**V9**
		**Week**	**0**	**1**	**2**	**4**	**6**	**8**	**16**	**26**	**52**
1	Clinical measures	PANSS	X	X	X	X	X	X	X	X	X
		CGI-S	X	X	X	X	X	X	X	X	X
		CGI-I	X	X	X	X	X	X	X	X	X
		CDSS	X	X	X	X	X	X	X	X	X
		GAF	X	X	X	X	X	X	X	X	X
2	Insight questionnaire	“My thoughts and feelings”	X					X		X	X
3	Prostaglandin synthesis (PGD2, PGE2)	Niacin flush skin test	X					X		X	
4	Cognitive function tests: Working memory and Executive functions	TMT Part A and B, and their alternate forms, i.e. Part C, D and Digit Span Forward and Backward	X					X		X	
	Verbal memory	CVLT	X					X		X	
	Visual memory	BVRT	X					X		X	
	Abstract thinking	Halstead Category Test	X					X		X	
	Verbal fluency	Semantic and Phonological	X					X		X	
5	Magnetic Resonance Imaging	Grey matter thickness - VBM	X							X	
		Fractional anisotropy – DTI	X							X	
6	Oxidative stress	Isoprostane 8-epi-PGF2alpha (plasma)	X					X		X	
	Antioxidative defense	Total plasma antioxidative capacity	X					X		X	
7	Neuronal plasticity	Plasma BDNF concentration	X					X		X	
8	Dietary questionnaire	Diet history questionnaire	X								
9	Metabolic syndrome traits	Plasma lipid profile	X					X		X	X
	(biochemical assessments)	Fasting Blood Glucose level	X					X		X	X
10	Metabolic syndrome traits	Body weight	X	X	X	X	X	X	X	X	X
		Waist circumference	X	X	X	X	X	X	X	X	X
	(clinical measures)	Blood pressure	X	X	X	X	X	X	X	X	X

**Abbreviations**: **PANSS** – Positive and Negative Syndrome Scale, **CGI-S** - Clinical Global Impressions - Severity, **CGI-I** - Clinical Global Impressions - Improvement, **CDSS** - Calgary Depression Scale for Schizophrenia, **GAF** – Global Assessment of Functioning, **PGD2** – Prostaglandin D2, **PGE2** - Prostaglandin E2, **TMT** – Trail Making Test Part, **CVLT** – California Verbal Learning Test, **BVRT** - Benton Visual Retention Test , **VBM** – Voxel Based Morphometry, **DTI** –Diffusion tensor Imaging, **8epiPGF2alpha** – plasma concentration of prostaglandin 8-epi-F-2alpha, **BDNF** – plasma concentration of brain derived neurotrophic factor.

### Statistical methods and analytic plan

#### Primary aim

The primary aim is to assess the efficacy of add-on n-3 PUFA therapy in reducing the severity of symptoms in schizophrenia patients after 26 weeks supplementation accompanying standard antipsychotic treatment. Hence, our primary outcome measure is the change observed over time on the total and subscale PANSS scores. The changes in the scores will be calculated by subtracting baseline from follow-up score at each follow-up assessment visit for each patient. Efficacy analyses will be performed using the modified intent-to-treat (ITT) population, which will consist of all randomized patients who will receive at least one daily dose of either placebo or active compound and have at least one efficacy evaluation on day seven or later. Observed case analysis will also be performed for the primary and secondary outcome measures.

Comparisons between treatment groups at baseline will be conducted using a one-way analysis of covariance, with intervention type as a term in the model and duration of untreated psychosis (DUP) as covariate. The distribution of socio-demographic characteristics in the two treatment arms will also be compared to assess the effectiveness of the randomization procedure.

To deal with missing values in our ITT sample, a multiple imputation technique will be used, in which a number of data sets are created, each with the missing values replaced by some plausible values. The imputation of the missing values is based on maximum likelihood and Bayesian procedures and is performed by appropriate algorithms. After the missing cases are completed by several sets of plausible values to create multiple completed datasets, standard complete-data procedures are applied to each completed dataset, and finally the multiple sets of results are combined to yield a single inference. The multiple imputation technique is considered superior to and less biased than the Last Observation Carried Forward (LOCF) [53,77].

The analysis of covariance (ANCOVA) with intervention type as term and baseline PANSS score as covariate will be used to assess the change from baseline in PANSS scores to week 8 and 26 separately. If the overall ANCOVA of the baseline-to-endpoint change score will be significant, pairwise comparison will be performed using Dunnett t test. Analyzes will be two-sided for both ANCOVA and Dunnett test with the type I error rate for rejecting null hypothesis set at 0.05. The same analyses will be repeated to assess the significance of differences in changes of CDSS, CGI and GAF scores between the study arms.

### Secondary aim

The secondary outcome of the study will be the efficacy of n-3 PUFA to prevent relapse in first-episode schizophrenia. Hence, the secondary null hypothesis will be that intervention is not different from placebo on the efficacy measure. Using the relapse rate as the secondary endpoint a one-sided log-rank test with an overall sample size of 82 subjects (41 per study arm) will achieve a 90.2 % power at a 0.05 significance level to detect a difference of 20 percentage points (regarded as clinically significant) in the relapse rate. Intention-to-Treat basis (ITT) will be used in analyses. Analysis of secondary endpoint will be by the Last Observation Carried Forward (LOCF) principle. The secondary outcome measure will be interpreted at 0.05 significance level.

### Safety and tolerability

All adverse events volunteered or observed during the study will be recorded, together with their severity and duration during all assessment visits. Tolerability will be measured with the Udvalg Kliniske Udersogelser Side Effect Rating Scale (UKU) [78], a semistructured interview for the assessment of the general side effects of psychotropic medication. The emergance and severity of extrapiramidal symptoms will be measured using the Simpson-Angus Scale (SAS) [79], and the Barnes akathisia scale (BAS) [80].

## Discussion

### Design weakness and limitations

The main limitation of the study is that it is not possible to measure the concentration of n-3 PUFA in the red blood cells of participants. Hence, it will not be possible to verify study medication adherence. To account for this, pill counts will be taken and data will be collected concerning medication adherence during every study visit. However, the randomization and blinding used in the study design indicates that the possible bias introduced at this level can be distributed equally between the study arms. However, the effect of the intervention may be decreased because of possible adherence problems, which can be difficult to detect.

### Methodological considerations

The current study has several strengths, including randomization of patients and blinding of both patients and staff. The used design (RCT) is the standard for the evaluation of efficacy of psychiatric treatments. The use of a longitudinal design also makes it possible to assess change and observe the postulated correlates of change. Another strength of the study is the composition of n-3 PUFA used, i.e. a 1.5:1 mixture of EPA and DHA, which has not yet been used in patients with first-episode schizophrenia. The dosage of PUFA supplementation is higher than in previous studies and low enough to assure safety of intervention. The length of the supplementation period is rather long in comparison with previous studies and equals six months, which enables both short and long-term efficacy assessment. The follow-up period after finishing supplementation makes it possible to assess the relapse rate after first-episode of schizophrenia. Finally the intervention is delivered to individuals with relatively short periods of the disease. Most of the above mentioned characteristics make the present study considerably different from the previous studies and constitute the advantages of the study design.

## Conclusion

It is intended to perform a randomized, double-blind, placebo-controlled, parallel-group single-center, six month, augmentation trial (RCT) of an intervention based on 2.2 g of EPA + DHA added on to an adjustable dose of antipsychotic medication in patients with first-episode schizophrenia. The main outcome measure will be the efficacy of n-3 PUFA in reducing symptom severity measured by means of PANSS. The secondary outcome measures constitute the following: (a) the efficacy of 6-month add-on therapy with n-3 PUFA in reducing the risk of relapse in first-episode schizophrenia patients during the first year of therapy; (b) the influence of n-3 PUFA on depressive symptoms, general functioning and level of insight. With its longitudinal design, the study aims also to observe correlates of the expected change, i.e. structural brain changes observed in MRI scans, oxidative stress and defense mechanisms as well as brain plasticity.

## References

[1] Assisi A, Banzi R, Buonocore C, Capasso F, Di Muzio V, Michelacci F (2006). Fish oil and mental health: the role of n-3 long-chain polyunsaturated fatty acids in cognitive development and neurological disorders. Int Clin Psychopharmacol.

[2] Singh M (2005). Essential fatty acids, DHA and human brain. Indian J Pediatr.

[3] Reddy RD, Keshavan MS, Yao JK (2004). Reduced red blood cell membrane essential polyunsaturated fatty acids in first episode schizophrenia at neuroleptic-naive baseline. Schizophr Bull.

[4] Peet M, Shah S, Selvam K, Ramchand CN (2004). Polyunsaturated fatty acid levels in red cell membranes of unmedicated schizophrenic patients. World J Biol Psychiatry.

[5] Arvindakshan M, Ghate M, Ranjekar PK, Evans DR, Mahadik SP (2003). Supplementation with a combination of omega-3 fatty acids and antioxidants (vitamins E and C) improves the outcome of schizophrenia. Schizophr Res.

[6] Khan MM, Evans DR, Gunna V, Scheffer RE, Parikh VV, Mahadik SP (2002). Reduced erythrocyte membrane essential fatty acids and increased lipid peroxides in schizophrenia at the never-medicated first-episode of psychosis and after years of treatment with antipsychotics. Schizophr Res.

[7] Carver JD, Benford VJ, Han B, Cantor AB (2001). The relationship between age and the fatty acid composition of cerebral cortex and erythrocytes in human subjects. Brain Res Bull.

[8] Amminger GP, Schafer MR, Klier CM, Slavik J, Holzer I, Holub M (2012). Decreased nervonic acid levels in erythrocyte membranes predict psychosis in help-seeking ultra-high-risk individuals. Mol Psychiatry.

[9] Hoen WP, Lijmer JG, Duran M, Wanders RJ, van Beveren NJ, de Haan L (2013). Red blood cell polyunsaturated fatty acids measured in red blood cells and schizophrenia: a meta-analysis. Psychiatry Res.

[10] Horrobin DF (1998). The membrane phospholipid hypothesis as a biochemical basis for the neurodevelopmental concept of schizophrenia. Schizophr Res.

[11] Horrocks LA, Farooqui AA (2004). Docosahexaenoic acid in the diet: its importance in maintenance and restoration of neural membrane function. Prostaglandins Leukot Essent Fatty Acids.

[12] Bach SA, de Siqueira LV, Muller AP, Oses JP, Quatrim A, Emanuelli T (2014). Dietary omega-3 deficiency reduces BDNF content and activation NMDA receptor and Fyn in dorsal hippocampus: implications on persistence of long-term memory in rats. Nutr Neurosci.

[13] Bondi CO, Taha AY, Tock JL, Totah NK, Cheon Y, Torres GE (2014). Adolescent behavior and dopamine availability are uniquely sensitive to dietary omega-3 fatty acid deficiency. Biol Psychiatry.

[14] Yao JK, Magan S, Sonel AF, Gurklis JA, Sanders R, Reddy RD (2004). Effects of omega-3 fatty acid on platelet serotonin responsivity in patients with schizophrenia. Prostaglandins Leukot Essent Fatty Acids.

[15] Ozyurt B, Sarsilmaz M, Akpolat N, Ozyurt H, Akyol O, Herken H (2007). The protective effects of omega-3 fatty acids against MK-801-induced neurotoxicity in prefrontal cortex of rat. Neurochem Int.

[16] Smesny S, Kunstmann C, Kunstmann S, Willhardt I, Lasch J, Yotter RA (2011). Phospholipase A(2) activity in first episode schizophrenia: associations with symptom severity and outcome at week 12. World J Biol Psychiatry.

[17] Rybakowski JK, Borkowska A, Czerski PM, Dmitrzak-Weglarz M, Hauser J (2003). The study of cytosolic phospholipase A2 gene polymorphism in schizophrenia using eye movement disturbances as an endophenotypic marker. Neuropsychobiology.

[18] Pae C, Yu H, Lee K, Kim J, Lee C, Lee S (2004). BanI polymorphism of the cytosolic phospholipase A2 gene may confer susceptibility to the development of schizophrenia. Prog Neuropsychopharmacol Biol Psychiatry.

[19] Kerr DS, Talib LL, Yamamoto VJ, Ferreira AS, Zanetti MV, Serpa MH (2013). Antipsychotic drugs decrease iPLA2 gene expression in schizophrenia. Schizophr Res.

[20] Rapoport SI, Rao JS, Igarashi M (2007). Brain metabolism of nutritionally essential polyunsaturated fatty acids depends on both the diet and the liver. Prostaglandins Leukot Essent Fatty Acids.

[21] Peet M, Brind J, Ramchand CN, Shah S, Vankar GK (2001). Two double-blind placebo-controlled pilot studies of eicosapentaenoic acid in the treatment of schizophrenia. Schizophr Res.

[22] Emsley R, Myburgh C, Oosthuizen P, van Rensburg SJ (2002). Randomized, placebo-controlled study of ethyl-eicosapentaenoic acid as supplemental treatment in schizophrenia. Am J Psychiatry.

[23] Fenton WS, Dickerson F, Boronow J, Hibbeln JR, Knable M (2001). A placebo-controlled trial of omega-3 fatty acid (ethyl eicosapentaenoic acid) supplementation for residual symptoms and cognitive impairment in schizophrenia. Am J Psychiatry.

[24] Peet M, Horrobin DF (2002). A dose-ranging exploratory study of the effects of ethyl-eicosapentaenoate in patients with persistent schizophrenic symptoms. J Psychiatr Res.

[25] Emsley R, Niehaus DJH, Koen L, Oosthuizen PP, Turner HJ, Carey P (2006). The effects of eicosapentaenoic acid in tardive dyskinesia: a randomized, placebo-controlled trial. Schizophr Res.

[26] Berger GE, Proffitt T, McConchie M, Yuen H, Wood SJ, Amminger GP (2007). Ethyl-eicosapentaenoic acid in first-episode psychosis: a randomized, placebo-controlled trial. J Clin Psychiatry.

[27] Fusar-Poli P, Berger G (2012). Eicosapentaenoic acid interventions in schizophrenia: meta-analysis of randomized, placebo-controlled studies. J Clin Psychopharmacol.

[28] McEvoy J, Baillie RA, Zhu H, Buckley P, Keshavan MS, Nasrallah HA (2013). Lipidomics reveals early metabolic changes in subjects with schizophrenia: effects of atypical antipsychotics. PLoS ONE.

[29] Pisano S, Gritti A, Catone G, Pascotto A (2013). Antipsychotic-induced dyslipidemia treated with omega 3 fatty acid supplement in an 11-year-old psychotic child: a 1-year follow-up. J Child Adolesc Psychopharmacol.

[30] Faghihi T, Jahed A, Mahmoudi-Gharaei J, Sharifi V, Akhondzadeh S, Ghaeli P (2012). Role of Omega-3 fatty acids in preventing metabolic disturbances in patients on olanzapine plus either sodium valproate or lithium: a randomized double-blind placebo-controlled trial. Daru.

[31] Barcelos RC, Silva BDM, Boufleur N, Reckziegel P, Muller LG, Pase C (2010). Effects of omega-3 essential fatty acids (omega-3 EFAs) on motor disorders and memory dysfunction typical neuroleptic-induced: behavioral and biochemical parameter. Neurotox Res.

[32] Sarsilmaz M, Songur A, Ozyurt H, Kus I, Ozen OA, Ozyurt B (2003). Potential role of dietary omega-3 essential fatty acids on some oxidant/antioxidant parameters in rats’ corpus striatum. Prostaglandins Leukot Essent Fatty Acids.

[33] Cardoso HD, Dos Santos Junior EF, de Santana DF, Goncalves-Pimentel C, Angelim MK, Isaac AR (2014). Omega-3 deficiency and neurodegeneration in the substantia nigra: involvement of increased nitric oxide production and reduced BDNF expression. Biochim Biophys Acta.

[34] Robinson D, Woerner MG, Alvir JM, Bilder R, Goldman R, Geisler S (1999). Predictors of relapse following response from a first episode of schizophrenia or schizoaffective disorder. Arch Gen Psychiatry.

[35] Liberman RP, Kopelowicz A (2005). Sustained remission of schizophrenia. Am J Psychiatry.

[36] Jaaskelainen E, Juola P, Hirvonen N, McGrath JJ, Saha S, Isohanni M (2013). A systematic review and meta-analysis of recovery in schizophrenia. Schizophr Bull.

[37] Lieberman JA, Stroup TS, Perkins DO (2006). The American Psychiatric Publishing textbook of schizophrenia.

[38] Alvarez-Jimenez M, Priede A, Hetrick SE, Bendall S, Killackey E, Parker AG (2012). Risk factors for relapse following treatment for first episode psychosis: a systematic review and meta-analysis of longitudinal studies. Schizophr Res.

[39] Linszen D, Dingemans P, Lenior M (2001). Early intervention and a five year follow up in young adults with a short duration of untreated psychosis: ethical implications. Schizophr Res.

[40] Emsley R, Chiliza B, Asmal L, Harvey BH (2013). The nature of relapse in schizophrenia. BMC Psychiatry.

[41] Alvarez-Jimenez M, Parker AG, Hetrick SE, McGorry PD, Gleeson JF (2011). Preventing the second episode: a systematic review and meta-analysis of psychosocial and pharmacological trials in first-episode psychosis. Schizophr Bull.

[42] Andreasen NC, Liu D, Ziebell S, Vora A, Ho B (2013). Relapse duration, treatment intensity, and brain tissue loss in schizophrenia: a prospective longitudinal MRI study. Am J Psychiatry.

[43] Emsley R, Chiliza B, Asmal L, Du Plessis S, Phahladira L, van Niekerk E, van Rensburg, Susan J, Harvey BH: A randomized, controlled trial of omega-3 fatty acids plus an antioxidant for relapse prevention after antipsychotic discontinuation in first-episode schizophrenia. Schizophr Res. 2014. 10.1016/j.schres.2014.06.004 24996507

[44] Nielsen J, Le Quach P, Emborg C, Foldager L, Correll CU (2010). 10-year trends in the treatment and outcomes of patients with first-episode schizophrenia. Acta Psychiatr Scand.

[45] van Bennekom L, Marc WH, Gijsman HJ, Zitman FG (2013). Antipsychotic polypharmacy in psychotic disorders: a critical review of neurobiology, efficacy, tolerability and cost effectiveness. J Psychopharmacol.

[46] Emsley R, Niehaus DJH, Oosthuizen PP, Koen L, Ascott-Evans B, Chiliza B (2008). Safety of the omega-3 fatty acid, eicosapentaenoic acid (EPA) in psychiatric patients: results from a randomized, placebo-controlled trial. Psychiatry Res.

[47] Harper KN, Hibbeln JR, Deckelbaum R, Quesenberry CP, Schaefer CA, Brown AS (2011). Maternal serum docosahexaenoic acid and schizophrenia spectrum disorders in adult offspring. Schizophr Res.

[48] Rogers LK, Valentine CJ, Keim SA (2013). DHA supplementation: current implications in pregnancy and childhood. Pharmacol Res.

[49] Zugno AI, Chipindo HL, Volpato AM, Budni J, Steckert AV, de Oliveira MB (2014). Omega-3 prevents behavior response and brain oxidative damage in the ketamine model of schizophrenia. Neuroscience.

[50] Cederholm T, Salem N, Palmblad J (2013). Omega-3 fatty acids in the prevention of cognitive decline in humans. Adv Nutr.

[51] Janssen CI, Kiliaan AJ (2014). Long-chain polyunsaturated fatty acids (LCPUFA) from genesis to senescence: the influence of LCPUFA on neural development, aging, and neurodegeneration. Prog Lipid Res.

[52] Jarema M (2011). Standards of pharmacotherapy of certain mental disorders. [Standardy leczenia farmakologicznego niektórych zaburzeń psychicznych].

[53] Schafer JL (1997). Analysis of incomplete multivariate data.

[54] Joy CB, Mumby-Croft R, Joy LA: Polyunsaturated fatty acid supplementation for schizophrenia. Cochrane Database Syst Rev. 2006:CD001257. 10.1002/14651858.CD001257 12804400

[55] Bentsen H, Osnes K, Refsum H, Solberg DK, Bohmer T (2013). A randomized placebo-controlled trial of an omega-3 fatty acid and vitamins E + C in schizophrenia. Transl Psychiatry.

[56] Amminger GP, Schafer MR, Papageorgiou K, Klier CM, Cotton SM, Harrigan SM (2010). Long-chain omega-3 fatty acids for indicated prevention of psychotic disorders: a randomized, placebo-controlled trial. Arch Gen Psychiatry.

[57] Farooqui AA (2012). n-3 fatty acid-derived lipid mediators in the brain: new weapons against oxidative stress and inflammation. Curr Med Chem.

[58] Begum G, Harvey L, Dixon CE, Sun D (2013). ER stress and effects of DHA as an ER stress inhibitor. Transl Stroke Res.

[59] Kuratko CN, Barrett EC, Nelson EB, Salem N (2013). The relationship of docosahexaenoic acid (DHA) with learning and behavior in healthy children: a review. Nutrients.

[60] Luchtman DW, Song C (2013). Cognitive enhancement by omega-3 fatty acids from child-hood to old age: findings from animal and clinical studies. Neuropharmacology.

[61] Sable PS, Kale AA, Joshi SR (2013). Prenatal omega 3 fatty acid supplementation to a micronutrient imbalanced diet protects brain neurotrophins in both the cortex and hippocampus in the adult rat offspring. Metabolism.

[62] Crupi R, Marino A, Cuzzocrea S (2013). n-3 fatty acids: role in neurogenesis and neuroplasticity. Curr Med Chem.

[63] Sivrioglu EY, Kirli S, Sipahioglu D, Gursoy B, Sarandol E (2007). The impact of omega-3 fatty acids, vitamins E and C supplementation on treatment outcome and side effects in schizophrenia patients treated with haloperidol: an open-label pilot study. Prog Neuropsychopharmacol Biol Psychiatry.

[64] Kay SR, Fiszbein A, Opler LA (1987). The positive and negative syndrome scale (PANSS) for schizophrenia. Schizophr Bull.

[65] Kay SR, Opler LA, Lindenmayer JP (1988). Reliability and validity of the positive and negative syndrome scale for schizophrenics. Psychiatry Res.

[66] Addington D, Addington J, Maticka-Tyndale E (1993). Assessing depression in schizophrenia: the Calgary Depression Scale. Br J Psychiatry Suppl..

[67] Addington D, Addington J, Maticka-Tyndale E, Joyce J (1992). Reliability and validity of a depression rating scale for schizophrenics. Schizophr Res.

[68] Addington J, Shah H, Liu L, Addington D (2014). Reliability and validity of the Calgary Depression Scale for Schizophrenia (CDSS) in youth at clinical high risk for psychosis. Schizophr Res.

[69] Jones SH, Thornicroft G, Coffey M, Dunn G (1995). A brief mental health outcome scale-reliability and validity of the Global Assessment of Functioning (GAF). Br J Psychiatry.

[70] Guy W (1976). The Clinical Global Impression Scale. ECDEU Assessment Manual for Psychopharmacology-Revised.

[71] Kokoszka A, Telichowska-Lesna A, Radzio R (2008). A questionnaire of insight into schizophrenia–“my thoughts and feelings”. Psychiatr Pol.

[72] Kotlicka-Antczak M, Pawelczyk T, Rabe-Jablonska J, Pawelczyk A: PORT (Programme of Recognition and Therapy): the first Polish recognition and treatment programme for patients with an at-risk mental state. Early Interv Psychiatry. 2014. 10.1111/eip.12146 24725353

[73] Leucht S, Davis JM, Engel RR, Kane JM, Wagenpfeil S (2007). Defining ‘response’ in antipsychotic drug trials: recommendations for the use of scale-derived cutoffs. Neuropsychopharmacology.

[74] Csernansky JG, Mahmoud R, Brenner R (2002). A comparison of risperidone and haloperidol for the prevention of relapse in patients with schizophrenia. N Engl J Med.

[75] Olivares JM, Sermon J, Hemels M, Schreiner A (2013). Definitions and drivers of relapse in patients with schizophrenia: a systematic literature review. Ann Gen Psychiatry.

[76] Smesny S, Berger G, Rosburg T, Riemann S, Riehemann S, McGorry P (2003). Potential use of the topical niacin skin test in early psychosis – a combined approach using optical reflection spectroscopy and a descriptive rating scale. J Psychiatr Res.

[77] He Y (2010). Missing data analysis using multiple imputation: getting to the heart of the matter. Circ Cardiovasc Qual Outcomes.

[78] Lingjaerde O, Ahlfors UG, Bech P, Dencker SJ, Elgen K (1987). The UKU side effect rating scale. A new comprehensive rating scale for psychotropic drugs and a cross-sectional study of side effects in neuroleptic-treated patients. Acta Psychiatr Scand Suppl.

[79] Simpson GM, Angus JW (1970). A rating scale for extrapyramidal side effects. Acta Psychiatr Scand Suppl.

[80] Barnes TR (1989). A rating scale for drug-induced akathisia. Br J Psychiatry.

